# Culture Conditions for Human Induced Pluripotent Stem Cell-Derived Schwann Cells: A Two-Centre Study

**DOI:** 10.3390/ijms24065366

**Published:** 2023-03-10

**Authors:** Zhong Huang, Rebecca Powell, Svenja Kankowski, James B. Phillips, Kirsten Haastert-Talini

**Affiliations:** 1 Institute of Neuroanatomy and Cell Biology, Hannover Medical School (MHH), 30623 Hannover, Germany; 2 Center for Systems Neuroscience (ZSN) Hannover, 30559 Hannover, Germany; 3 Department of Pharmacology, University College London (UCL) School of Pharmacy, 29-39 Brunswick Square, London WC1N 1AX, UK; 4 UCL Centre for Nerve Engineering, UCL, London WC1H 0AL, UK

**Keywords:** human-induced pluripotent stem cells, human Schwann cell precursors, human immature and mature Schwann cells, culture conditions

## Abstract

Adult human Schwann cells represent a relevant tool for studying peripheral neuropathies and developing regenerative therapies to treat nerve damage. Primary adult human Schwann cells are, however, difficult to obtain and challenging to propagate in culture. One potential solution is to generate Schwann cells from human induced pluripotent stem cells (hiPSCs). Previously published protocols, however, in our hands did not deliver sufficient viable cell numbers of hiPSC-derived Schwann cells (hiPSC-SCs). We present here, two modified protocols from two collaborating laboratories that overcome these challenges. With this, we also identified the relevant parameters to be specifically considered in any proposed differentiation protocol. Furthermore, we are, to our knowledge, the first to directly compare hiPSC-SCs to primary adult human Schwann cells using immunocytochemistry and RT-qPCR. We conclude the type of coating to be important during the differentiation process from Schwann cell precursor cells or immature Schwann cells to definitive Schwann cells, as well as the amounts of glucose in the specific differentiation medium to be crucial for increasing its efficiency and the final yield of viable hiPSC-SCs. Our hiPSC-SCs further displayed high similarity to primary adult human Schwann cells.

## 1. Introduction

Nerve regeneration is a relevant research topic since nerve damage can be severe and existing treatment therapies have limited functional results [1]. In comparison with the central nervous system, peripheral nerves may heal after trauma, but successful regeneration depends on many factors [2]. Following nerve damage, axons distal to the injury site deteriorate; consequently, the primary aim of the regeneration process is axonal regeneration and target reinnervation. Following a transection injury, directed regrowth of axons requires their migration through two distinct environments, namely, the bridge region of new tissue that forms at the wound site, and the distal stump, which remains attached to the target tissues but requires remodeling to create an environment conducive to axonal regrowth. Axonal migration within these two settings needs unique, complicated multicellular mechanisms that operate to guide and sustain axonal regeneration [3,4]. Schwann cells are peripheral glial cells and crucial key players in both contexts for directing the regeneration process. This is accomplished by an extraordinary reprogramming process in which quiescent and highly specialized adult Schwann cells are reprogrammed into proliferating repair Schwann cells that coordinate the regeneration response and play specific functions in the various areas of the peripheral nerve [3,5]. Lineage analyses revealed the efficacy of this mechanism, with all mature Schwann cells downstream of the damage reentering the cell cycle within days after the injury [6]. Consequently, peripheral nerves have an intrinsic capacity for regeneration; however, the repair ability of Schwann cells is reduced with time following damage, in part due to the breakdown of glial-axonal communication as peripheral nerve fibres deteriorate [2,7,8,9]. Peripheral neuropathies or traumatic peripheral nerve injuries, therefore, often result in chronic denervation of target tissue and this is, to a considerable part, due to Schwann cell dysfunction limiting functional regeneration outcomes for patients [9,10].

Non tissue engineered (i.e., cell-free) nerve guidance conduits (NGCs), e.g., used for bridging “critical length” peripheral nerve tissue defects, also perform less efficiently compared with nerve autografts in supporting healing and functional recovery, in part due to their lack of cellular support [11]. Thus, instead of using them as a simple hollow nerve bridge, NGCs may be more promising as a delivery platform for growth-promoting substrates such as nerve and glial growth factors, tiny segments of peripheral nerve, or simply Schwann cells themselves [10,12]. In fact, it has been demonstrated that autologous nerve-derived Schwann cells within NGCs promote faster and more effective nerve regeneration than empty NGCs alone [13,14]. However, identifying a suitable supply of adult human Schwann cells (ahSCs) for therapeutic purposes remains a major challenge for translating cell therapy approaches into the clinic [5,10,15,16,17,18].

Additionally, the majority of research published to date has examined nerve regeneration using rodent cells and tissues, which may not be fully comparable to human. Given that the ultimate goal is human clinical translation, human derived cells are a valuable tool for research as well as being the likely optimal choice for therapy. With human induced pluripotent stem cells (hiPSCs) developing as a viable resource for regenerative medicine, human iPSC-derived SCs (hiPSC-SCs) are being investigated as a possible cell source for peripheral nerve repair approaches [1,15,19,20]. The possibility of being able to obtain clinical-grade hiPSCs, and to adhere to Good Manufacturing Practice (GMP) regulations, provides the opportunity of a standardised treatment protocol [21].

During embryonic development, Schwann cells originate from the migratory, transient population of neural crest cells [22], which migrate to the periphery and differentiate into Schwann cell precursors (SCPs) before maturing into non-myelinating and myelinating Schwann cells [23,24,25]. Therefore, methods of differentiating hiPSCs to hiPSC-SCs pass through an intermediate progenitor cell stage—either neural crest stem cells or hiPSC-derived SCPs [24]. Most studies focus on differentiating neural crest cells or neural crest stem cells as middle step cells for the final production of Schwann cells or Schwann cell-like cells for cell therapy [15]. These protocols typically require more time to scale up compared with the differentiation via a SCP step [25], and the purity of the finally differentiated cells is limited. Recent studies show that through a specified SCP step, hiPSCs can be converted into Schwann cells on a larger scale and in a reduced time period [26]. Moreover, it has been reported that human iPSC-derived Schwann cell precursors (hiPSC-SCPs) can be stably expanded for at least 35 passages without a loss of differentiation potential, even after cryopreservation [25]. Importantly, it has been reported that only about a week is necessary for hiPSC-SCPs to gain functional hiPSC-SCs competence [25,27]. Those characteristics will be of benefit when subsequently employed to form cellular substrates for nerve tissue engineering [1,25,27,28]. Previously published protocols show the feasibility of generating hiPSC-SCs; however, they mostly resulted in the limited yield and viability of hiPSC-SCs in vitro [29]. Furthermore, for application in an advanced therapy medicinal product (ATMP), such as a tissue engineered construct, GMP-grade cells and reagents have to be used where these options are available. Such constructs typically require millions of cells to be seeded per cm of length [30], therefore the relatively low yields achieved with early protocols for hiPSCs differentiation make this an important limiting factor [25].

Therefore, our teams from two separate but internationally collaborating laboratories at University College London (UCL) and Hannover Medical School (MHH), have modified the previously published protocol [25] in parallel attempts. The protocols used were not 100% identical; indeed, in clinical practice, protocols at different locations tend to differ slightly. The UCL method, for example, was kept antibiotic free throughout due to the potential impact of antibiotic use on cell proliferation and differentiation [31,32,33], while in the MHH protocol, antibiotic agents were added throughout the differentiation process from hiPSCs to hiPSC-SCs. 

The overall aim for both teams was to reproducibly produce differentiated and viable hiPSC-SCPs or hiPSC-immature SCs, respectively, and hiPSC-SCs at quantities not reached with previously published protocols [25]. By comparing our protocols, we have identified relevant common parameters for modification to address the limitations mentioned above, contributing valuable new understanding to this area of research and development.

## 2. Results

In this study, the two collaborating laboratories from UCL and MHH worked with two different populations of hiPSCs and detected similar parameters to be relevant for modification towards differentiating higher numbers of viable hiPSC-SCs from hiPSC-SCPs or hiPSC-immature SCs within a week. 

**Figure 1:**
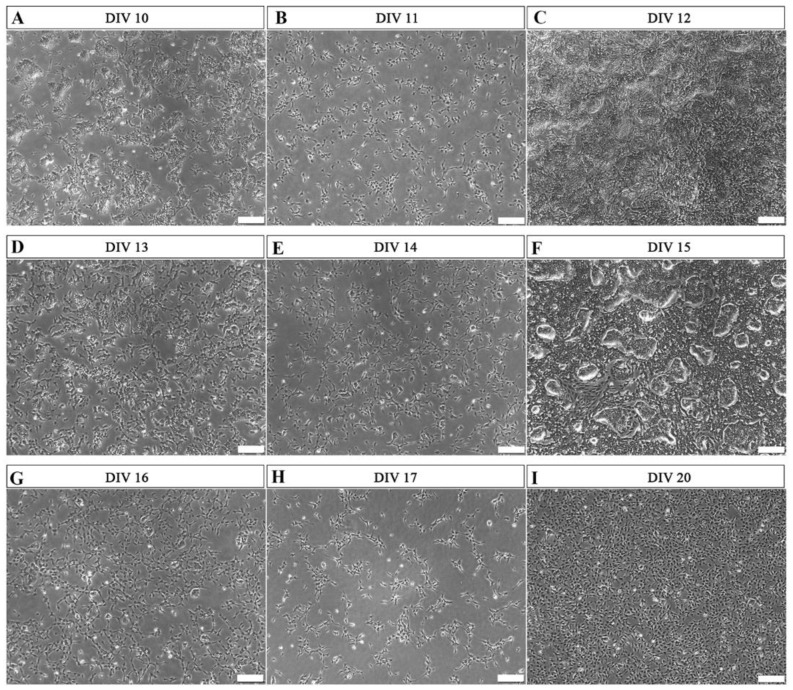
Representative phase-contrast photomicrographs of cells undergoing differentiation from hiPSCs to hiPSC-immatureSCs. Matrigel coating was used during this period. (**A**,**D**,**G**) show the morphology of the differentiating cells at 60–70% confluence, after 10 days in vitro (DIV 10) (**A**), DIV 13 (**D**), and DIV 16 (**G**), respectively. After this, cells were alternatively passaged to a lower cell density of 2 × 10^5^ per well in a 6-well plate ((**B**)—DIV 11, (**E**)—DIV 14, (**H**)—DIV 17) or continuously cultured, without splitting, in high cell density ((**C**)—DIV 12, (**F**)—DIV 15, (**I**)—DIV 20). DIV = days in vitro. Scale bar: 200 µm.

*At MHH*, the overall differentiation process was initiated when human induced pluripotent stem cells (hiPSCs) were cultured in Schwann cell precursor differentiation medium (SCPDM) onto matrigel-coated plates. On day 10 (DIV 10, Figure 1A), cells already presented with a multipolar shape growing outside of the initally formed clusters. 

Two different cell-density conditions were compared from this stage onward. After reaching 60–70% confluence (Figure 1A,D,G), the cells were either passaged with a density of 2 × 10^5^ cells per well in a 6-well plate (Figure 1B,E,H), or alternatively maintained in the non-splitted high cell density culture without passaging (Figure 1C,F,I). In the latter condition, the morphology of cell clusters reduced during the differentiation process (Figure 1C,F), and on day 20 (Figure 1I), the hiPSCs were nearly fully differentiated into a stage with certain characteristics different from the commonly defined precursor stage. The MHH cells were already more mature as will be demonstrated below. Therefore, we name these cells immature Schwann cells (hiPSC-immatureSCs), representing an interim stage between hiPSC-SCPs and hiPSC-SCs.

**Figure 2:**
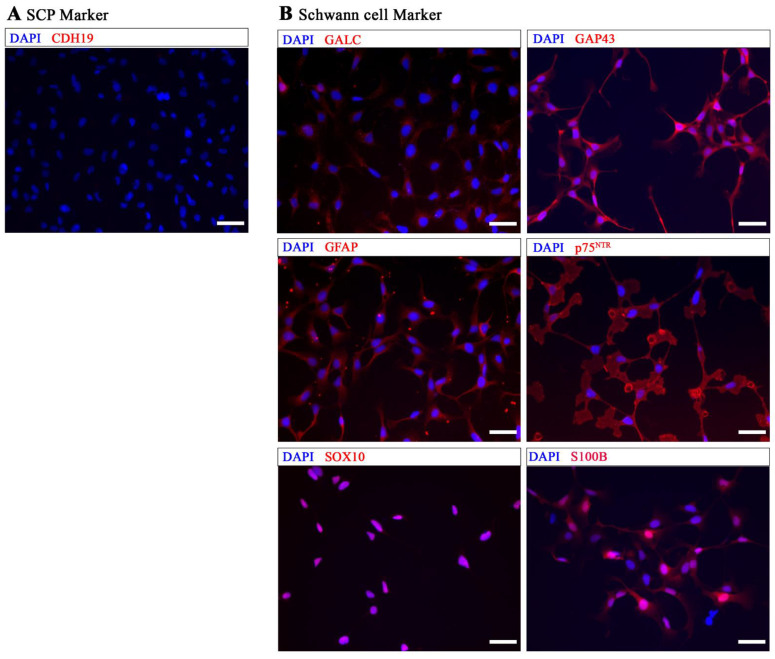
Immunocytochemical analysis of MHH-hiPSC-immatureSCs. (**A**) shows that hiPSC-immatureSCs were negative for CDH19. (**B**) shows positive staining for Schwann cell markers GALC, GAP43, GFAP, p75NTR, SOX10, and S100B. Cell nuclei were stained with DAPI (Blue). Scale bar: 50 µm.

Immunocytochemistry (ICC) was used to further characterize these differentiated cells at DIV 20 using antibodies against CDH19, SOX10, p75^NTR^, GAP43, GFAP, S100B, and GALC (Figure 2). The ICC results demonstrated that the cells were already negative for CDH19, a marker commonly considered to be specific for SCPs (Figure 2A), and positive for Schwann cell markers SOX10, p75^NTR^, GAP43, GFAP, S100B, and GALC (Figure 2B). 

More infromation about the phenotypic changes of the cells during the differentiation phase is illustrated in the supplementary material. After 18 days under MHH differentiation conditions (SCPDM + antibiotics), slender or triangular-shaped hiPSC- immatureSCs were obtained from hiPSCs (Figure S1A). The hiPSC-immatureSCs were further grown in antibiotic supplemented SCPDM with the concentration of neuregulin 1 (NRG1) increased from 50 ng/mL to 100 ng/mL. Due to their preserved proliferation activity and a high degree of homogeneity, hiPSC- immatureSCs were then grown at a cell density of 2–10 × 10^5^ per well in a 6-well plate (Figure S1A–C) and subcultured when they achieved confluence. The cells were quite stable and homogeneous in their morphology when cultured at the same cell density for at least 11 passages, even after interim cryopreservation. Analysis of sister cultures in ICC validated the negative expression of the marker CDH19 and positive expression of Schwann cell markers SOX10, S100B, and p75^NTR^ (Figure S1D–G). In a preliminary test, these hiPSC-immatureSCs further demonstrated significantly increased expression levels of Schwann cell marker genes, e.g., *SOX10*, *GAP43*, *PMP22*, *PLP1* and *MPZ*, from passage 2 to passage 6 (Figure S1H). At the same time, we also see a significant increase in *TFAP2A* and *CDH19*, which are commonly discussed to be markers for the precursor stage, however, are also highly expressed by our control primary human Schwann cells, see below. 

Interestingly, when cultured at a different cell density, we found that hiPSC-immatureSCs displayed quite different morphologies. The cells grown at a lower cell density (2.5 × 10^4^ cells per well, 24-well plate) showed a more flat and variable cell morphology, and cells grown at a higher cell density (5 × 10^4^ cells per well, 24-well plate) changed into a small round morphology (Figure S2). Immunostaining for the Schwann cell markers SOX1O, S100B and p75^NTR^ were positive, regardless of the initial hiPSC-immatureSC cell density seeded (Figure S2).

**Figure 3:**
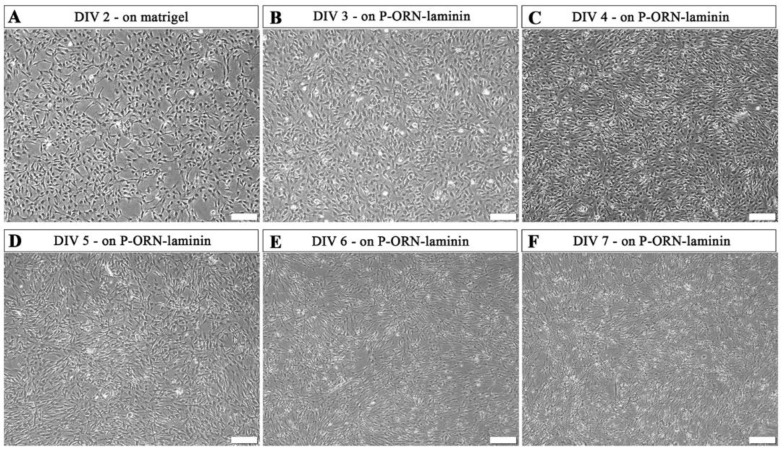
Representative phase-contrast photomicrographs of hiPSC-immatureSCs undergoing differentiation to hiPSC-SCs. hiPSC-immatureSCs cultured in MHH-SCDM after 2 days on matrigel coating ((**A**), DIV 2) and after the coating was changed to poly-L-ornithine (P-ORN)-laminin (**B**–**F**). Cell numbers continuously increased with a lowering pace and started showing an elongated morphology at DIV 3 (**B**) and DIV 4 (**C**). After this, cells visibly differentiated more instead of further increasing their density at DIV 5 (**D**). At DIV 6 (**E**) and DIV 7 (**F**), most of the cells showed an elongated morphology. Differentiation was coming to an end at DIV 7 (**F**), when undifferentiated cells were not observed within the cultures. Scale bar: 200 µm. MHH-SCDM = Schwann cell differentiation medium as composed by the MHH team.

At MHH, final hiPSC-immatureSCs differentiation into hiPSC-SCs was initiated with a cell density of 2 × 10^5^ per well in a matrigel-coated 6-well plate applying platelet-derived growth factor-BB (PDGF-BB), retinoic acid (RA), forskolin, and NRG1 in Schwann cell differentiation medium (MHH-SCDM). At DIV 2, the cells were proliferating without displaying morphological alterations (Figure 3A). After two days on the matrigel-coated plate, the cells were subcultured onto poly-L-ornithine (P-ORN)-laminin-coated cell culture surfaces, where they further proliferated, as shown for DIV 3 (Figure 3B) and DIV 4 (Figure 3C), while a small number of cells already displayed a differentiated elongated shape. On DIV 5 (Figure 3D), hiPSC-immatureSCs showed a rather reduced increase in cell density but an induced differentiation towards the elongated Schwann cell phenotype. Finally, the morphology of the hiPSC-immatureSC derived cells changed into an elongated shape at DIV 6 and 7 (Figure 3E,F), indicating that the majority of cells now represents the mature stage of hiPSC-SCs. At DIV 7 to DIV 9, the MHH hiPSC-SCs demonstrate a viable population growing in a mean density of 3.8 × 10^5^ cells/cm^2^ (quantification from *n* = 3 independent differentiation trials.

When we compared the hiPSC-SCs derived by the modified MHH protocol with the published characteristics of hiPSC-SCs derived from a previous protocol [25], we could find a clear improvement in the differentiation efficiency and viability of the cells (Figure S3). When using the original protocol [25] in our hands, we found many cells still growing in cell clusters, which were immunonegative for the Schwann cell markers S100B and p75^NTR^ (Figure S3), thus revealing a poor differentiation efficiency compared to the cells derived from the modified MHH protocol (Figure S3).

**Figure 4:**
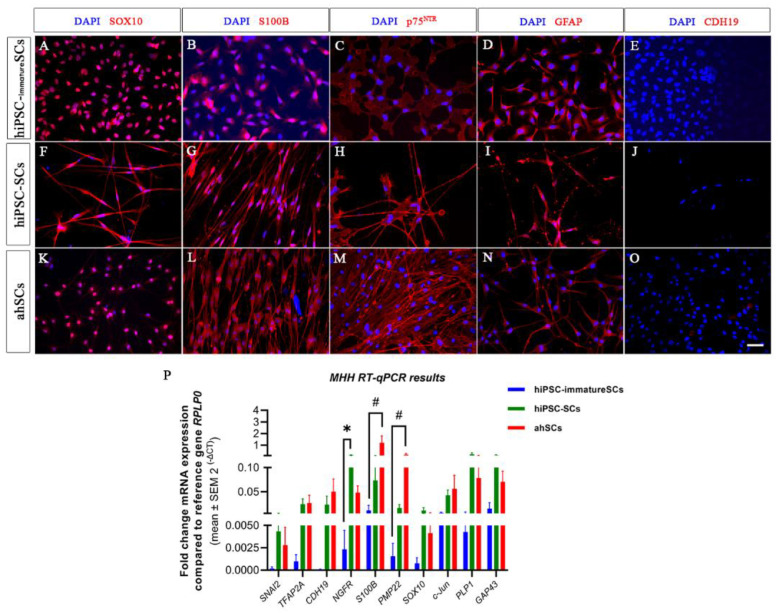
Comparative immunocytochemistry on hiPSC-immatureSCs, hiPSC-SCs and adult human Schwann cells (ahSCs). Schwann cell markers SOX10, S100B, p75^NTR^, and GFAP were detected in hiPSC-immatureSCs (**A**–**E**), hiPSC-SCs (**F**–**H**,**I**,**J**) and ahSCs (**K**–**O**). All cells were negative for CDH19 (**E**,**J**,**O**). Cell nuclei were stained with DAPI (Blue). Scale bar: 50 µm. (**P**) MHH results of quantitative RT-qPCR for *SNAI2, TFAP2A, CDH19, NGFR, S100B, PMP22, SOX10, c-Jun*, *PLP1*, and *GAP43* in hiPSC-immatureSCs, hiPSC-SCs and ahSCs. Values were normalised to the reference gene *RPLP0*. Statistical analysis with non-parametric Kruskal–Wallis test followed by Dunn’s multiple compansons test, * *p* < 0.05 hiPSC-immatureSCs vs. hiPSC-SCs; # *p* < 0.05 hiPS-immatureSCs vs. ahSCs. *n* = 3 cell cultures.

To better understand the characteristics of the hiPSC-SCs derived from the modified MHH protocol, we further used primary adult human Schwann cells (ahSCs) as a control in ICC for the Schwann cell markers, SOX10, S100B, p75^NTR^, GFAP, and CDH19, commonly discussed to be a marker for the precursor stage (Figure 4). ICC results show that hiPS-SCs, ahSCs, and hiPSC-immatureSCs were all immunopositive for SOX10 (Figure 4A,F,K), S100B (Figure 4B,G,L), p75^NTR^ (Figure 4C,H,M), and GFAP (Figure 4D,I,N). However, SOX10 was detectable in the cell nuclei in the hiPSC-immatureSCs (Figure 4A), while hiPSC-SCs and ahSCs displayed its presence both in the nuclei and cytoplasm (Figure 4F,K). The latter finding may relate to the actual function of non-myelinting Schwann cells in the in vitro conditions, because there are indications from the literature that SOX10 translocation into the cytoplasm is related to a decreased myelinating function in mature Schwann cells [34]. All cell types analysed, hiPSC-immatureSCs (Figure 4E), hiPSC-SCs (Figure 4J), and ahSCs (Figure 4O), were detected to be immunonegative for the CDH19 antigen.

For further proving the identity of MHH hiSCP-SCs as “real” Schwann cells, we additionally performed a comparative gene analysis with primary adult human Schwann cells (ahSCs). We conducted RT-qPCR analysis for *SNAI2, TFAP2A, CDH19, NGFR, S100B, PMP22, SOX10, c-Jun, PLP1,* and *GAP43* to examine the expression of key marker genes associated with different stages of Schwann cell development. Gene expression levels of *SNAI2, TFAP2A, CDH19, SOX10, c-Jun, PLP1,* and *GAP43* were not significantly different between hiPSC-immatureSCs, hiPSC-SCs, and ahSCs (Figure 4P). The mRNA expression level of *NGFR* gene, reported to be upregulated in repair Schwann cells [11], was significantly higher in hiPSC-SCs than in hiPSC-immatureSCs, indicating the more progressed differentiation. The mRNA expression level of the myelinating Schwann cell marker gene *S100B* and *PMP22* [11], however, was significantly higher in adult human Schwann cells compared with hiPSC-immatureSCs, but not significantly different between hiPSC-SCs and primary adult human Schwann cells (Figure 4P). The latter finding indicates that, from hiPSC-SCs to fully functional adult human Schwann cells, the cells will have to undergo further modifications in their gene expression profiles, which also depend upon the specific environment the Schwann cells will have to adapt to [35]. 

**Figure 5:**
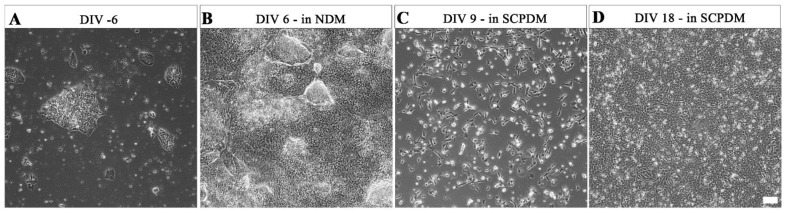
Phase contrast micrographs of morphological changes over the differentiation process in antibiotic free media from (**A**) hiPSCs; (**B**) at DIV 6 in NDM; (**C**) at DIV 9 in SCPDM; (**D**) hiPSC-SCPs at DIV 18 in SCPDM. hiPSCs (**A**) were plated on vessels coated with vitronectin, with the coating changed to Geltrex™ prior to the addition of NDM (**B**–**D**). Scale bar = 100 µm. NDM = neural differentiation medium; SCPDM = Schwann cell precursor differentiation medium.

*At UCL*, hiPSCs were maintained in distinct colonies (Figure 5A). These hiPSCs began to proliferate rapidly once the media was changed from Essential 8 flex media to antibiotic free neural differentiation media (NDM, Figure 5B). Between 6 days in vitro (DIV 6) and then DIV 18 in antibiotic free SCPDM, the cells became evenly dispersed over the Geltrex^TM^ coated culture vessels and densely populated the surface (Figure 5C,D). The hiPSC-SCPs (precursor stage) has been determined by [25] to be reached at DIV 18 in antibiotic free SCPDM (Figure 5D).

Again, hiPSC-SCP plating density into SCDM was found to be critical for successful differentiation to hiPSC-SCs in 7 days (Figure S4). At a high-density of 8 × 10^5^ per 10 cm plate (14,104/cm^2^), few cells formed the typical bipolar morphology of Schwann cells (Figure S4A), with most cells unchanged from the precursor stage (Figure 5D). At the lower density of 10^5^ per 10 cm plate (1763/cm^2^, Figure S4B), the cells adopted a bipolar morphology and looked similar to primary adult human Schwann cells (Figure S4C). However, this density is rather low for long-term survival of the differentiated hiPSC-SCs and proliferation for use in downstream applications. Increasing plating densities were assessed to find a balance between Schwann cell morphology and survival (Figure S5). Increasing the initial hiPSC-SCP plating density in SCDM 8-fold improved survival and maintained the bipolar morphology (Figure S5D).

**Figure 6:**
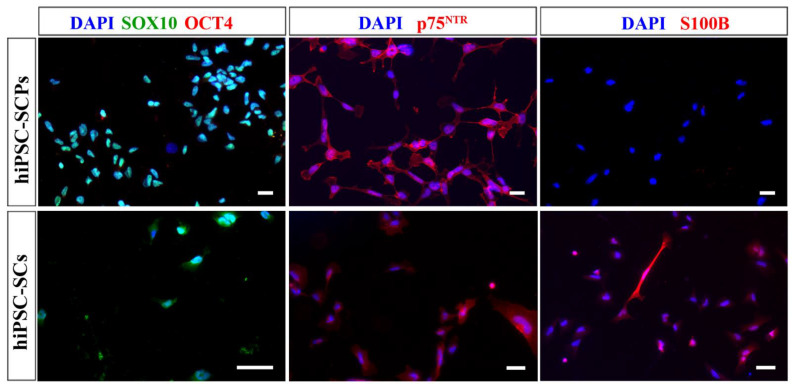
Immunocytochemistry images of UCL hiPSC-SCPs and hiPSC-SCs from low-density plated hiPSC-SCPs. Both cell types were positive for SOX10 (green) and p75^NTR^ (red). Only hiPSC-SCs were positive for S100B (red) while both were negative for OCT4 (red). DAPI was used to stain the nuclei (blue). Scale bar = 20 µm (hiPSC-SCPs), 50 µm (hiPSC-SCs).

The proteins chosen to characterise the differentiated UCL hiPSC-SCPs and hiPSC-SCs were SOX10, OCT4, p75^NTR^, and S100B. Comparing differentiated hiPSC-SCPs to the hiPSC-SCs, both express the Schwann cell-lineage marker SOX10 (Figure 6). Both cell types were positive for p75^NTR^ (Figure 6) and S100B staining was much brighter in hiPSC-SCs compared to hiPSC-SCPs (Figure 6). Especially because the UCL hiPSC-SCPs do not show robust S100B antigen expression but the early MHH hiPSC-derived cells do, we propose that the interim stage represented by the UCL cells is indeed the precusor stage, while the interim stage represented by the MHH cells is already the immature Schwann cell stage. 

**Figure 7:**
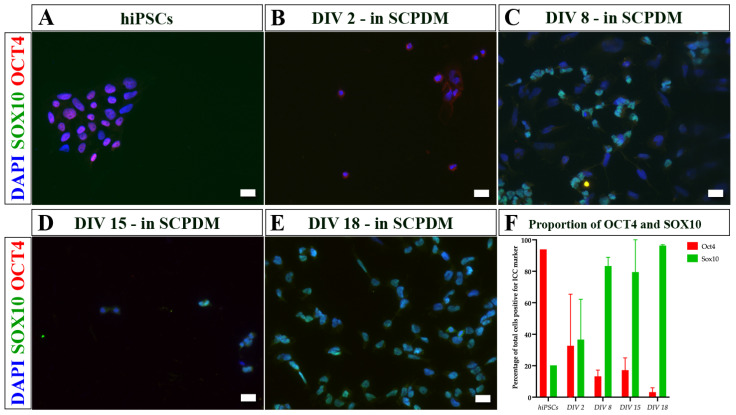
Immunocytochemistry images of (**A**) hiPSCs and (**B**–**E**) differentiating cells at different incubation times in antibiotic free SCPDM. (**B**) DIV 2, (**C**) DIV 8, (**D**) DIV 15, and (**E**) DIV 18. Cell nuclei were stained with DAPI (blue). (**F**) Proportion of total cells positive for OCT4 and SOX10 over time in SCPDM from two differentiation inductions. SOX10 (a marker for the Schwann cell lineage) is shown in green, increasing in proportion of the population over time, while OCT4 (a marker for pluripotency) is shown in red, decreasing over time. Scale bar = 20 µm. SCPDM = Schwann cell precursor differentiation medium.

As the cells differentiated from hiPSCs to hiPSC-SCPs (Figure 7), the change from hiPSCs growing in colonies to more dispersed cells occured early on once the media was changed from Essential 8 Flex media to antibiotic free NDM and SCPDM (Figure 7B). Furthermore, the proportion of cells positive for OCT4 reduced while the number of SOX10 positive cells increased over time, reaching close to 100% at DIV 18 in SCPDM (Figure 7E,F). 

**Figure 8:**
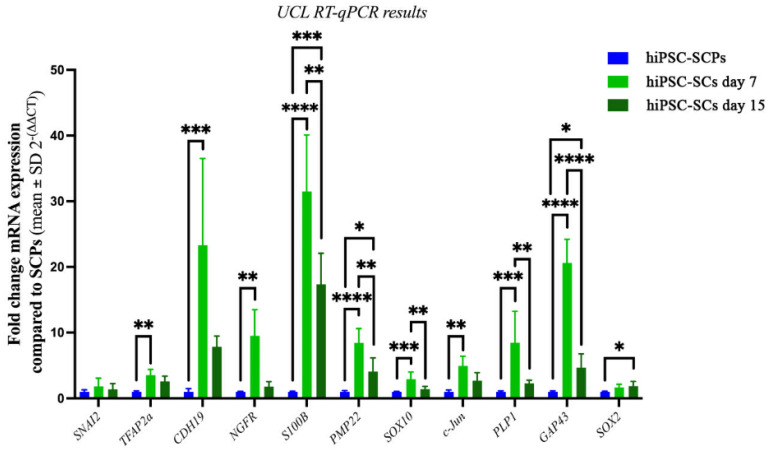
UCL RT-qPCR results showing fold change in mRNA expression in hiPSC-SCs after 7 (light green) or 15 days in SCDM (dark green) compared with hiPSC-SCPs (blue), which was set at 1. Shapiro–Wilks normality test followed by either one-way ANOVA with Tukey’s post-comparison test, * *p* < 0.02, ** *p* < 0.004, *** *p* < 0.0005, **** *p* < 0.0001 or Kruskal–Wallis with Dunn’s multiple comparison post-hoc test for non-normal distributions (*SNAI2, TFAP2A, CDH19, NGFR, c-Jun*), * *p* = 0.039, ** *p* <0.003, *** *p* = 0.0003. *n* = 3 in duplicate, mean ± SD. All were normalised to the reference genes *TBP* and *RPS18*.

For further characterization of the hiPSC derived cells at UCL, mRNA was extracted from hiPSC-SCPs at DIV 18 in SCPDM and differentiated hiPSC-SCs at DIV 7 and DIV 15 in SCDM. The fold change in mRNA expression in both hiPSC-SCs cell populations was compared with hiPSC-SCPs (Figure 8). Several genes associated with immature or mature Schwann cells were significantly upregulated in the differentiated hiPSC-SCs at DIV 7: nerve growth factor receptor (*NGFR*), S100 Calcium binding protein B (*S100B*), peripheral myelin protein 22 (*PMP22*), and cadherin 19 (*CDH19*). The latter has been detected in rat Schwann cell precursors, but is also present in our primary adult human Schwann cells (see Figure 4P above). Proteolipid protein 1 (*PLP1*) and SRY-Box transcription factor 10 (*SOX10*) were also upregulated and expressed by both immature Schwann cells and SCPs. Transcription factor AP-2 alpha (*TFAP2A)* is typically associated with early neural crest cells and SCPs but was also upregulated in hiPSC-SCs compared to hiPSC-SCPs at DIV 7 in SCDM. Once again, we detected similar expression levels for *TFAP2A* in MHH hiPSC-SCs and ahSC (see Figure 4P, above). Upregulated too in UCL hiPSC-SCs compared to UCL hiPSC-SCPs at DIV 7 in SCDM was the transcription factor *c-Jun*, which is key in reprogramming mature Schwann cells to repair Schwann cells. There were no significant differences in Snail Family Transcriptional Repressor 2 (*SNAI2*) or *SOX2* between hiPSC-SCPs and DIV 7 hiPSC-SCs. *SOX2* was, however, significantly upregulated in DIV 15 hiPSC-SCs. Overall, following the UCL procedures, the cells taken at DIV 15 had a greater similarity in gene expression pattern to the hiPSC-SCPs rather than DIV 7 hiPSC-SCs. Genes that were significantly upregulated between DIV 15 hiPSC-derived cells and hiPSC-SCPS were *S100B*, *PMP22*, *c-Jun* and *GAP43*. 

Morphological observation indicated an evident change in the UCL hiPSC-derived cells at DIV 15 in SCDM where some cells retained the bipolar shape of Schwann cells while others seemed to revert to the hiPSC-SCP morphology, growing with closer association to each other (Figure S6B).

## 3. Discussion

In an effort to contribute to the supply of functional hiPSC-SCs at scale and in a shorter amount of time, we concentrated on identifying relevant culture conditions for the differentiation period that, if carefully considered, will help to overcome the constraint of low cell yield of hiPSC-SCs from hiPSCs. This attempt was strengthend by the fact that we have independently modified the original protocol published in 2017 [25] in two collaborationg laboraties, *MHH* and *UCL*. 

We discovered that passaging the cells during the differentiation period from hiPSCs to hiPSC-SCPs or hiPSC-immatureSCs is advisable to be performed when confluence is only about 60–70%, which takes about 2–3 days using *the MHH protocol*. This avoids the formation of cell clumps, which in turn, we found to considerably reduce the differentiation efficiency. Rock-inhibitor (Y-27632) was used to help the survival of the cells by supporting cell attachment on the plate for the first 12–24 h after passaging during the whole differentiation process from hiPSCs to hiPSC-immatureSCs. The differentiated hiPSC-immatureSCs can be expanded at least 11 passages without loss of their differentiation potential, even after cryopreservation. 

Likewise, in the antibiotic free *UCL procedure*, focusing on the plating density of the hiPSC-SCPs when transitioning to UCL-SCDM from SCPDM resulted in the differentiation of hiPSC-SCs that survived and proliferated for up to 2 weeks. This allows the *UCL protocol* to be used to generate differentiated human Schwann cells for tissue engineering applications, which require millions of cells to produce an implantable nerve construct [30,36,37,38]. 

Further, the plating density was investigated as primary human Schwann cells are highly sensitive to density, displaying contact-dependent proliferation [39]. Casella et al. previously found 1.8–3.5 × 10^3^ cells per cm^2^ to be optimal for Schwann cell proliferation, which did result in cells of the typical bipolar Schwann cell shape [1]. However, the addition of the differentiation medium UCL-SCDM—the bulk of which is low glucose Dulbecco’s Modified Eagle Medium (DMEM)—could cause greater stress on the cells resulting in cell death and decreasing the plating density too low for optimal survival and proliferation. Increasing the plating density 8-fold in the *UCL protocol* increased the yield of differentiated Schwann cells without impacting the morphology and expression of Schwann cell markers.

Similarly, *at MHH*, using DMEM with low glucose (1 g/L) as the basic medium according to the previously published study [25], performed not as well as the use of Advanced DMEM/F12 with high glucose (4.5 g/L). Furthermore, a density of 2 × 10^5^ cells per well in a 6-well plate was used at the beginning of the *MHH differentiation procedure* from hiPSC-immatureSCs to hiPSC-SCs. After two days, these cells were passaged onto a poly-L-ornithine (P-ORN)-laminin coated 6-well plate with a density of 4 × 10^5^ cells per well. This was performed because we assumed that less cell-to-cell contact contributes to the differentiation from precursor cells, while differentiated hiPSC-SCs required more cell-to-cell contact for maintaining in proliferation. This fits well with our experience from the culturing of adult human Schwann cells [40]. 

The coating also matters during the hiPSC-SCPs or hiPSC-immatureSCs to hiPSC-SCs differentiation process. The use of Geltrex™ as a coating in the *UCL procedure* showed that the protocol based on the previously published protocol in 2017 [25] can be used with GMP-ready reagents, which is beneficial for the use of Schwann cells in translational research. 

Schwann cells differentiated under antibiotic free conditions with the *UCL protocol* and maintained beyond DIV 7 in SCDM, however, appeared to show a pattern of de-differentiation when comparing hiPSC-SCs from DIV 7 and DIV 15. A significant increase in *SOX2*, a pluripotency marker [41], as well as significant decreases in genes associated with mature Schwann cells such as *NGFR, S100B* and *PMP22,* could also indicate a mixed population of differentiation stages, which is supported by the morphological changes seen by DIV 15 in UCL-SCDM. The Schwann cell phenotype is dependent on the presence of NRG1 [42], which is provided by UCL-SCDM. However, the availability of NRG1 will be reduced as the cells proliferate. Long-term maintenance of the differentiated hiPSC-SCs could be improved by culturing with forskolin, which induces cAMP activation to enable the cells to respond to NRG1 more effectively [20,43]. 

Using the *MHH procedure*, some different observations were made. For the first two days, matrigel coated culture plates were used and then replaced with P-ORN-laminin coated culture plates. This procedure was chosen because cells on the matrigel coating in differentiation medium differentiated well, but also easily died thereafter, while on the P-ORN-laminin coating, differentiated cells continued to proliferate well. Under these conditions, after four days of differentiation, the hiPSC-SCs slowly began to exhibit the elongated bipolar morphology of Schwann cells that we also detect, when culturing adult human Schwann cells on P-ORN-laminin coated surfaces. After 7 days of differentiation, almost all *MHH hiPSC-SCs* displayed the elongated bipolar morphology of primary adult human Schwann cells. Instead of the dedifferentiation observed in the UCL protocol at DIV 15, the MHH hiPSC-SCs showed reduced viability after DIV 10.

Publications so far, targeting the potential of iPSC-derived SCs in peripheral nerve repair (either from human or animal sources), evaluated their cells in rodent autograft repair models (the “gold standard” treatment for long gap peripheral nerve injuries). However, no standardised control cells have been used to verify the stem cell-derived cell identities [25,28,44,45]. Therefore, it is unclear how similar the differentiated iPSCs investigated were to adult Schwann cells. With regard to a future translation into clinical use, comprehensive analysis of the properties of differentiated iPSCs is an important step. 

Tackling this point in our study, it was important to show similarity in morphology to primary adult human Schwann cells alongside gene and protein expression patterns. To our knowledge, this is the first study to compare the features of obtained hiPSC-SCs to human Schwann cells.

The *UCL culture* of hiPSCs in SCDPM initially resulted in a rapid decrease of the pluripotency marker Octamer-binding transcription factor 4 (OCT4) [41,46,47] alongside an increase in SOX10, which is switched on in the glial cell lineage [23]. SOX10 is also important in the next stages of differentiation as it allows cells to respond to neuregulin (NRG1) via receptor tyrosine-protein kinase (ErbB-3) [48,49]. NRG1 is essential for Schwann cell development and maturation [42,50] and is present in both SCPDM and SCDM, with double the concentration in SCDM [25]. The *UCL ICC results* show both differentiated hiPSC-SCPs and hiPSC-SCs expressed p75^NTR^ (NGFR) on the membrane, which is associated with immature Schwann cells and precursors [27,51,52,53], whereas only the hiPSC-SCs expressed S100B, which is only found in immature to mature Schwann cells [52,54]. These results match the pattern seen in the *UCL RT-qPCR*. Looking at the genes upregulated at DIV 7 in hiPSC-SCs in SCDM compared to hiPSC-SCPs or hiPSC-immature SCs, the majority are associated with immature to mature Schwann cells. *CDH19* has previously been described to be associated with only the Schwann cell precursor stage in rats [49,52] but has also been found in human Schwann cells [51,55], so the increase in mRNA expression levels seen in our differentiated Schwann cells was expected. The *MHH RT-qPCR results* also show that CDH19 was upregulated at DIV 7 in MHH hiPSC-SCs and primary adult human Schwann cells. *MHH ICC * results, however, did reveal that all cell stages analysed (hiPSC-immatureSCs, -SCs, and ahSCs) were immunonegative for the CDH19 antigen. This indicates that CDH19 antigen expression may indeed only be present in the precursor stage, while mRNA levels are continuously high also in the other stages and CDH19 may not be an exclusive marker for the precursor stage. This is supported by recent analysis of the mouse peripheral nerve transcriptome [56]. Another important transcription factor gene expressed in the differentiated hiPSC-SCs was *c-Jun*. This transcription factor is not only important for the repair phenotypic switch [18] but also for the formation of the guiding Bands of Büngner—and without *c-Jun*, reinnervation cannot occur after injury of mouse sciatic nerves [7,57]. Implanting cells that express *c-Jun* could improve outcomes in peripheral nerve injury repair as they should more closely match the phenotype of the host repair Schwann cells.

Despite the need for additional in vivo characterisation, the *MHH team characterised* their hiPSC-immatureSCs s and hiPSC-SCs by using primary adult human Schwann cells as a powerful control. *The MHH hiPSC*-immatureSCs, as an essential intermediate product during the differentiation from hiPSCs to hiPSC-SCs, displayed a very stable cell morphology and proliferative capacity in vitro, as shown by the ICC and RT-qPCR in the results; therefore, could be a good source for further differentiation into hiPSC-SCs in a short period (one week in our hands). Since repair Schwann cells mimic an embryonic Schwann cell phenotype and SCPs are an expandable cell population, some authors postulate that a pure SCP population may be far better for possible future clinical applications compared to mature myelinating or non-myelinating Schwann cells [11]. Interestingly, our results show that hiPSC-SCPs were not only immunopositive for the SCP markers SOX10, p75^NTR^, and GAP 43, but also for the Schwann cell specific markers S100B, GFAP, and GALC. Further, the *MHH RT-qPCR results* revealed no significant differences in mRNA expression levels for the marker genes *SNAI2, TFAP2A, CDH19, S100B, SOX10, c-Jun, PLP1,* and *GAP43*, which are related to different stages of Schwann cell development; except for the finding of a significantly lower expression of myelination related gene *PMP22* compared to adult human Schwann cell levels. Based on these results, we defined the early hiPSC-derived cells with the MHH protocol as hiPSC- immatureSCs. In contrast the UCL protocol did, in the same differentiation period, deliver cells that were still S100B immunonegative and, therefore, represent indeed the precursor state. Others have previously demonstrated that rat neural crest cell-derived SCPs implanted into a rat sciatic nerve crush lesion improved the outcome of nerve regeneration and functional restoration over that achievable with implantation of finally differentiated Schwann cells [58]. In vitro co-culture of SCPs with injured dorsal root ganglion (DRG) neurons indicated higher mRNA expression levels for the genes *MBP*, *P0*, *PMP22* and *PLP* in SCPs, which could thereby promote DRG neurons to restore soma survival and axon growth [59]. Since the survival of injected cells is an essential aspect of cell therapy approaches for successful nerve regeneration, the positive results obtained after SCP implantation may likely depend on the strong proliferative and survival capacity of SCPs in vivo [58]. 

Therefore, further studies are needed for a deeper characterization and functional analysis of our hiPSC-SCPs.

## 4. Materials and Methods

### 4.1. Culturing of Human Induced Pluripotent Stem Cells

#### 4.1.1. By the Hannover Medical School Team-MHH

The human induced pluripotent stem cells (hiPSCs) used in this study were from the hiPSC line Phoenix (hHSC_Iso4_ADCF_SeV-iPS2; MHHi001-A) derived from CD34+ human cord blood haematopoietic stem cells [60]. The hiPSCs were transgene-free and isolated from cord blood samples by gradient centrifugation and magnetic-activated cell sorting. Reprogramming was performed using non-integrating CytoTune™ Sendai reprogramming vectors (Invitrogen, Georgia, Germany) delivering OCT4, SOX2, c-MYC and KLF-4 transcription factors. The culture plates were coated with growth factor-reduced matrigel (BD Bioscience, Heidelberg, Germany) with a final concentration of 10 µL/mL in Dulbecco’s Modified Eagle Medium (DMEM) for 45 min in the 37 ℃, 5% CO_2_ incubator ready for use. The hiPSCs were then cultured onto growth factor reduced matrigel-coated 6-well plates with mTeSR™1 (STEMCELL Technologies, Köln, Germany) medium in the 37 ℃ incubator with 5% CO_2_. Medium was changed every day with 1.5 mL mTeSR™1 and the colonised hiPSCs were routinely passaged when they reached 60–70% confluency (usually 5–7 days after seeding the cells). hiPSCs were passaged at a ratio of 1:20–50 with 266 U/mL pre-warmed collagenase IV. For the detaching of the colonised hiPSCs, medium was discarded and cells washed once with PBS. In total, 266 U/mL pre-warmed collagenase IV at 1 mL/10 cm^2^ was added and incubated for 40 min at 37 °C in a 5% CO_2_ incubator until a surrounding membrane appeared. Cell colonies were washed out softly using the supernatant and 1 mL mTeSR1/well. Medium and supernatant were transferred into a 15 mL falcon and centrifuged for 5 min at the speed of 100× *g*. The cell pellet was resuspended, and cells seeded with a ratio of 1:20–50 on the 6-well plate with mTeSR™1.

#### 4.1.2. By the University College London Team-UCL

‘GMP-ready’ hiPSCs from CD34+ peripheral blood cells from a donor in New Zealand were obtained from the UK Cell and Gene Therapy Catapult. hiPSCs were thawed to 6-well plates and expanded in T25–75 cm^2^ flasks coated in vitronectin at 0.5 µg/cm^2^ in Essential 8 Flex media (Thermo Fisher Scientific, Loughborough, UK). In total, 1 mL/10 cm^2^ Essential 8 Flex media was exchanged every weekday, with a double volume added on Fridays to allow feed-free weekends. hiPSCs were passaged at a ratio of 1:4 once confluency was at or greater than 80%, usually every 4–5 days. The cell culture vessels were washed with PBS once to remove dead cells before adding the passaging agent—0.5 mM EDTA at 1 mL/10 cm^2^. Cell culture vessels were incubated at room temperature for up to 5 min until the hiPSCs in colonies were bright around the edges of the cells across the whole surface. The EDTA was aspirated, and media used to rapidly dislodge cells from the culture vessel, ensuring the cells remained in clumps (rather than single cells). Cell suspensions were gently pipetted into a 15 mL falcon tube and divided into the appropriate number of new culture vessels. Flasks or plates were transferred to a humidified 5% CO_2_, 37 °C incubator.

For cryopreservation the passaging protocol was followed until the cell resuspension was collected. At this point, hiPSCs were counted by mixing a 10 µL aliquot of cells with 10 µL Trypan blue, forcefully pipetting the cells to break up large clumps before plating 10 µL of the solution onto a haemocytometer. The desired number of cells was transferred to a 15 mL falcon tube and centrifuged at 200× *g* for 5 min. hiPSC pellets were resuspended in CryoStor^®^ at a density of 1 × 10^6^ cells per ml, with 1 mL per cryovial. Cryovials were transferred into an insulated container for controlled-rate freezing to −80 °C for 24 h before moving to −150 °C or liquid nitrogen storage.

### 4.2. Culturing of Primary Adult Human Schwann Cells—Both MHH and UCL

Primary adult human Schwann cells (ahSCs) were isolated from the remnants of peripheral nerve transplants taken during reconstructive surgery in vivo from 13 to 60 year-old male or female donors according to a previously published protocol [61]. To enrich the primary ahSCs, purification was performed by employing the cold jet technique published previously [40]. In brief, cells were grown to 70–90% confluency on a 6-well plate before the wells were flushed each with 1 mL ice-cold phosphate-buffered saline (PBS), which had to be applied carefully (drop-by-drop) and then immediately aspirated. Schwann cells were detached from the fibroblast ground layer by applying an additional stream of 1 mL ice-cold melanocyte growth medium into each well and flushing appropriately several times, rinsing especially those spots with high cell density. Cell suspensions, mainly Schwann cells, were then transfered in 15 mL falcons. The detachment should be monitored with phase-contrast microscopy, and repeated several times. After centrifugation (at 1000 rpm for 5 min at RT) (Heraeus Multifuge X 3 F Centrifuge, Thermo Scientific, Schwerte, Germany), the enriched ahSCs were cultivated, as described previously [62], on a poly-L-ornithine (P-ORN)-laminin-coated 6-well plate at 37 °C and 5% CO_2_ in an ahSC-specific culture medium containing 1% bovine serum albumin (BSA). Seeding density was 3.5 × 10^5^ cells/well on a 6-well plate (3.7 × 10^4^ cells/cm^2^). ahSCs culture medium containing 2 µM forskolin, 10 ng/mL human FGF, 5 g/mL BPE-26, 2.5 ng/mL insulin, 10 nM recombinant human NRG1, 1% Pen/Strep and 1% amphotericin B in melanocyte growth medium. After 24 h, the culture medium was changed into ahSC culture medium and changed thereafter every 2–3 days. On average, 3–4 purifications were performed until the cells were pure enough for further analysis. The purity of the ahSCs was identified by using immunocytochemistry on a 24-well plate against intracellular calcium-binding protein anti-S100B or cell surface molecule p75^NTR^.

### 4.3. Differentiation from hiPSCs to hiPSC-SCPs or hiPSC-immatureSCs

#### 4.3.1. By the Hannover Medical School Team—MHH

hiPSCs cultured onto growth factor-reduced matrigel-coated 6-well plates for 5–7 days were detached by using 1 mL 1× accutase per well (STEMCELL Technologies, Colone, Germany) at 37 °C, in 5% CO_2_ atmosphere for 4–5 min until the hiPSCs presented as floating single cells. The enzyme reaction was then stopped by adding warm serum-containing DMEM (DMEM + 10% foetal bovine serum (FBS)). The cell pellet was collected by centrifugation at 1000 rpm for 5 min at RT (Heraeus Multifuge X3F Centrifuge, Thermo Scientific, Germany) and seeded in a density of 2 × 10^5^ cells per well in a 6-well plate in neural differentiation medium (NDM) with additional antibiotic agents, 1% Pen/Strep and 1% amphotericin B. NDM contains 1x N2, 1x B27, 0.005% BSA, 2 mM GlutaMAX, 0.11 mM beta-mercaptoethanol, 3 mM CT99021, and 20 mM SB431542 (PeproTech EC Ltd., London, UK) in Advanced DMEM/F12 and Neurobasal media (1:1 mix) (GIBCO, Thermo Fisher Scientific, Waltham, MA, USA). Medium used in this period needed to be freshly changed every day for the first six days. After 6 days of differentiation, 50 ng/mL neuregulin-1 (NRG1) was added with NDM, which was called Schwann cell precursor differentiation media (SCPDM), with additional antibiotic agents, 1% Pen/Strep and 1% amphotericin B. The cells were routinely dissociated with 1 mL 1× accutase per well in a 6-well plate upon reaching 60–70% confluence. CHIR 99021, SB 431542 and NRG1 were added freshly during each medium exchange, due to their short half-life period. The human iPSC-derived immature Schwann cells (hiPSC-immatureSCs) were differentiated after approximately 18 days of culturing under these conditions. Rock-inhibitor (Y-27632) (Abcam Cambridge UK) was used every time for the first 12–24 h after passaging during the whole differentiation process from hiPSCs to hiPSC-immatureSCs. By seeding with a cell density of 5–10 × 10^5^ on each well in a 6-well plate at the beginning, the hiPSCs- hiPSC-immatureSCs could be cultured in a maintaining medium by simply changing the SCPDM supplementation from 50 ng/mL NRG1 to 100 ng/mL NRG1. The cells could be passaged again once they reached a confluence of 90–100% or cultured without passaging for at least ten days with a maintaining medium change every two days.

#### 4.3.2. By the University College London Team—UCL

In the second modified protocol, hiPSCs were initially expanded to T25 flasks before starting differentiation. hiPSCs were differentiated to human Schwann cell precursors (hiPSC-SCPs) and human Schwann cells (hiPSC-SCs) using an adapted version of the protocol published by Kim et al. [25]. hiPSCs were first passaged as before but to flasks coated with 0.8 mL/10 cm^2^ Geltrex Ready-to-Use or Geltrex LDEV-Free hESC-qualified Reduced Growth Factor Basement Membrane Matrix (diluted 1:100 in DMEM/F12). hiPSCs on Geltrex^TM^ were incubated for 24 h in Essential 8 Flex media before changing the media to antibiotic free NDM. After 6 days of differentiation, the medium was changed to SCPDM. SCPDM was changed every day, and differentiating cells were passaged on reaching a confluency of 90% or more with Accutase as the passaging agent. Due to the absence of serum, a PBS wash was not required prior to adding Accutase, however used media was removed. Cell culture vessels were incubated with Accutase for 3–5 min at 37 °C before dislodging the cells by adding an equal volume of PBS. The cell suspension was pipetted into a 15 mL Falcon tube and centrifuged at 200× *g* for 5 min and the cell pellet resuspended in the appropriate volume of media for passaging. If the cells were to be counted, the cell pellet was resuspended in 5–10 mL PBS and 10 µL mixed with an equal volume of Trypan blue before adding 10 µL onto a haemocytometer. Cells were frozen at 2 × 10^6^ cells per ml of CryoStor^®^ in each cryovial. Cryovials were transferred to −80 °C for 24 h before moving to −150 °C or liquid nitrogen storage.

To ensure factors did not degrade in the media, a base media was made up with 1x N2, 1x B27, 0.005% BSA, 2 mM GlutaMAX and 0.11 mM beta-mercaptoethanol to 50 mL total volume. The volumes of other factors (CHIR99021, SB431542 and NRG1) were added per flask as required for either NDM or SCPDM.

After 18 days in UCL-SCPDM, hiPSC-SCPs were determined to have been produced as shown in [25].

### 4.4. Differentiation from hiPSC-SCPs or hiPSC-immatureSCs to hiPSC-SCs

#### 4.4.1. By the Hannover Medical School Team—MHH

For hiPSC-derived Schwann cells (hiPSC-SCs) differentiation, hiPSC-immatureSCs were seeded with a density of 2 × 10^5^ cells per well on a 6-well plate with matrigel coating for two days and then passaged onto P-ORN-laminin coating with a cell density of 4 × 10^5^ per well on a 6-well plate for two more days. Schwann cell differentiation medium (MHH-SCDM), containing 22% DMEM (4.5 g/L glucose), 10% FBS, 4 µM forskolin, 100 nM retinoic acid (RA), 10 ng/mL recombinant human platelet-derived growth factor-bb (PDGF-BB) and 200 ng/mL NRG1 with additional antibiotic agents (1% Pen/Strep and 1% amphotericin B) in Advanced DMEM/F12 was used for four days. After four days, forskolin and RA were omitted from the MHH-SCDM and the cells were cultured for another two days. Then, an adult human Schwann cell culture medium was used with a medium change every two to three days and the hiPSCs-SCs were maintained in culture for further processing. To quantify the outcome from the MHH protocol, we counted the number of viable cells in *n* = 3 representative phasecontrast photomicrographs taken from the DIV 7–DIV 9 cultures of hiPSC-SCs (see, for example, Figure 3F or Figure S3 *hiPSC-SCs from MHH modified protocol*). To calculate the cell density/cm^2^ (in 4× magnification), we counted, in each photomicrograph, all visible cell bodies in 8 randomly selected grid boxes with a grid area of 60,000 µm^2^ each. For setting grid parameters and for semiautomated counting, we used ImageJ (version 1.48; National Institutes of Health, Bethesda, MD, USA).

#### 4.4.2. By the University College London Team—UCL

To differentiate hiPSC-SCPs to hiPSC-SCs at UCL, antibiotic free media was changed to SCDM as directed by [25], containing 1% FBS, 200 ng/mL human heregulin beta-1, 4 µM forskolin, 100 nM retinoic acid and 10 ng/mL PDGF-BB in DMEM/low glucose. After 3 days, the media was changed to SCDM without forskolin or retinoic acid. After 2 further days, the media was changed to UCL-SCDM without PDGF-BB. A base media, UCL-SCDM of 50 mL DMEM/low glucose with 1% FBS, was made up and other factors added per flask as needed. To successfully differentiate hiPSC-SCPs to hiPSC-SCs, hiPSC-SCPs were plated at a density of 1763 cells/cm^2^ (10^5^ in 10 cm plate). To increase the numbers, this was increased 8-fold to 14,104 cells/cm^2^.

### 4.5. Immunocytochemistry

#### 4.5.1. By the Hannover Medical School Team—MHH

Cells were fixed by incubation in 4% paraformaldehyde in PBS for 20 min. For the ahSCs and hiPSC-SCPs, fixation was conducted after three days in culture on a 24-well plate. hiPSC-SCs were fixed directly after a differentiation period of seven days on a 6-well plate. The fixed cells were washed three times with PBS for 5 min. Blocking solutions for avoiding unspecific antibody binding were used according to the location of the target antigen. For the membrane staining, the blocking solution was PBS with 2% horse serum and 2% normal goat serum (NGS, GIBCO, Thermo Fisher Scientific, Waltham, MA, USA), while for cellular or nuclear staining 0.3% Triton-X-100 (Sigma-Aldrich Chemie GmbH, Taufkirchen, Germany) with 3% NGS in PBS was used as the blocking solution. Blocking was performed for 20 min at RT and the cells were then incubated overnight at 4 °C with primary antibodies, as shown in Supplementary Table S1. After washing three times with PBS at RT, Alexa 488 labelled goat anti-rabbit, Alexa 488 labelled goat anti-mouse secondary antibody, or Alexa 555 labelled goat anti-mouse secondary antibodies (all 1:500 in PBS, all Invitrogen, ThermoFisher Scientific, Waltham, MA, USA) (1:500 in PBS) were added for 1 h at RT in the dark. All cell nuclei were counterstained with 4′,6-Diamidine-2′-phenylindole dihydrochloride (DAPI 1:1000 in PBS) for 10 min at RT in the dark. Using an IX70 fluorescent microscope and cellSens software version 3.4 (Olympus Europa SE & Co. KG, Hamburg, Germany), images were aquired (Olympus Europa SE & Co. KG, Hamburg, Germany) for analysis.

#### 4.5.2. By the University College London Team—UCL

Images from the UCL protocol were obtained by the following process, with antibodies detailed in Supplementary Table S2. Coverslips were coated with 500 µL Geltrex (or vitronectin for hiPSCs) in 24-well plates and 10^4^ cells were seeded during passaging. Coverslips were washed twice with 1 mL DPBS and fixed for 15 min at room temperature in 500 µL 4% paraformaldehyde, before washing three times in 1 mL PBS. Coverslips were kept in PBS until immunocytochemistry (ICC) was performed. For intracellular protein detection, cells were permeabilised by incubating in 100 µL 0.5% Triton-X for 15 min at room temperature and washed three times by dipping in PBS. All cells, both for detecting intra- and extracellular protein, were blocked for 15 min by incubating at room temperature in 110 µL 5% serum (animal serum corresponded to the species in which the secondary antibody was raised); for intracellular staining, this was diluted in 0.5% Triton-X. Coverslips were washed three times in PBS before adding primary antibodies (100 µL per coverslip) alongside 5% blocking serum, diluted in 0.5% Triton-X for intracellular stains and incubating at 4 °C overnight, or 3 h at room temperature. Coverslips were then washed three times in PBS as before. Coverslips were incubated in 100 µL of the appropriate secondary antibody at 1:200 dilution for 45 min at room temperature before incubating with DAPI at 1:1000 dilution.

### 4.6. RNA Extraction and Real-Time Quantitative Reverse Transcription Polymerase Chain Reaction (Real-Time RT-qPCR)

#### 4.6.1. By the Hannover Medical School Team—MHH

The RNA extraction was conducted according to the manufacturer’s guidelines (RNeasy Plus Mini Kit, Qiagen, Hilden, Germany). For the ahSCs and hiPSC-SCPs, RNA extraction was conducted after three days in culture, and the RNA extraction of hiPSC-SCs was conducted directly after the 7 day differentiation period from hiPSC-SCPs. The harvested RNA was then used for reverse transcription applying iScriptTM cDNA Synthesis Kit (BioRad, Hercules, CA, USA) according to the manufacturer’s guidelines. The RT-qPCR was performed as recently described [63,64]. In brief, the reaction mixture was prepared in a MicroAmp reaction plate (Applied Biosystems^TM^, Foster City, CA, USA) containing 5 µL diluted cDNA, 2 µL forward and reverse primer (1.75 µM each) and 7 µL Power SYBR green PCR Master Mix (Applied Biosystems^TM^). The RT-qPCR was performed on a StepOnePlus thermocycler (Applied Biosystems^TM^): PCR was conducted for 40 cycles (15 s 95 °C and 1 min 60 °C) after an initial 10 min step of 95 °C. C_T_ values were calculated with StepOne-software version 2.3 using a constant cycle threshold of 0.2. Relative amount of transcript in cDNA levels was calculated and normalised to the reference gene (*RPLP0*). The primer sequences are given in Supplementary Table S3. *MHH* performed this experiment in biological triplicates and technical duplicates.

#### 4.6.2. By the University College Team—UCL

RT-qPCR at UCL was performed using the primers detailed in Supplementary Table S4. Cells were counted and washed twice in PBS before removing the PBS and immediately freezing the cell pellet at −80 °C. To extract RNA, the RNeasy Plus Mini Kit and protocol was followed using 350 µL buffer RLT, eluting in 30 µL nuclease-free water and reusing the eluate to extract a higher concentration of RNA. RNA was extracted from hiPSCs, hiPSC-SCPs at DIV 18 and hiPSC-SCs at DIV 7 and DIV 15 in SCDM. A benchtop centrifuge set at 9800× *g* was used for all spin steps. RNA concentration and 260/280 ratio was measured using the Take3 plate application on the Synergy™ HTX Multi-Mode Microplate Reader (BioTek^®^, Agilent, Santa Clara, CA, USA), using RNAse-free water as a blank and a volume of 1.5 µL. 0.2 µg RNA in 10 µL nuclease-free water was incubated for 5 min at 75 °C on the SimpliAmp Thermal Cycler (Applied Biosystems™) as a pre-RT step before adding to a mix of 4 µL nuclease-free water, 4 µL buffer and 2 µL enzyme (from GoScript™ Reverse Transcriptase) per sample on ice and continuing with reverse transcription using the SimpliAmp Thermal Cycler (Applied Biosystems™). cDNA was diluted 1 in 10 in nuclease-free water in 50 µL total and kept at −20 °C. For qPCR, 2 µL of each sample was added to a mix of 2.5 µL forward primer, 2.5 µL reverse primer, 10 µL SYBR Green MasterMix and 3 µL nuclease-free water in duplicate and run with a qPCR programme on QuantStudio 3 Real-Time PCR System (Applied Biosystems™). Fold change was calculated by comparing the change in CT between each gene of interest and the mean of the two reference genes *RPS18* and *TBP*. The hiPSC-SCPs were used as the control group to which the hiPSC-SCs were compared, with hiPSC-SCP values set at 1 for visualisation and statistical analysis in GraphPad Prism 9, version 9.5.0. 

### 4.7. Statistical Analysis

*MHH* RT-qPCR statistical analysis was performed using Excel Version 2016 (Microsoft Cooperation, Redmond, WA, USA) and GraphPad Prism version 9.2.0 (GraphPad Software Inc., La Jolla, CA, USA). Normal distribution of all data sets was examined with Shapiro –Wilk test. Non-parameter Kruskal–Wallis test with Dunn’s multiple comparisons post-hoc test was applied. All results are presented as percentages or mean ± SEM as indicated in the figures.

Statistical significance was defined and indicated as follows: one symbol = *p* < 0.05; two symbols = *p* < 0.01; three symbols = *p* < 0.001.

*UCL* RT-qPCR statistical analysis was performed using GraphPad Prism version 9.5.0 (GraphPad Software Inc.). Normal distribution of the fold change in gene expression was tested using Shapiro–Wilks. Data with normal distributions were analysed using one-way ANOVA with Tukey’s post-hoc comparison and non-normal distributions analysed using Kruskal–Wallis test with Dunn’s multiple comparison post-hoc test. Fold change was calculated by comparing the CT values between the hiPSC-SC groups to hiPSC-SCP group before using 2^−(ΔΔCT)^. The hiPSC-SCP fold change was then set to 1 and hiPSC-SC adjusted accordingly for graphical visualisation, with the mean ± standard deviation. 

## 5. Conclusions

The modified *UCL protocol* allows the successful differentiation of both hiPSC-SCPs and hiPSC-SCs from a GMP-ready hiPSC line under antibiotic free conditions. Both cell types can be expanded for use in downstream applications with differentiated hiPSC-SCs surviving up to 15 days. The stability of the differentiated cells is a drawback but may be improved by maintaining cultures at a reduced confluency to prevent dedifferentiation and the appearance of a mixed population. Further work would be focused on long-term survival as well as assessing any impact of cryopreservation on Schwann cell phenotype to ensure these are an option for translational research.

With the modified *MHH protocol*, we have solved the limitations of low cell gain of hiPSC-SCs from hiPSCs. The characterization of the derived hiPSC-SCs revealed, for the first time to our knowledge, their high similarity to primary adult human Schwann cells. The similarity was detected not only in terms of cell morphology and immunocytochemistry for Schwann cell specific markers but also in the expression of various Schwann cell related genes. 

Our collaborative work revealed hiPSC-SC seeding densities during propagation, SCDM glucose levels, and the cell culture surface coating, to be relevant parameters for generating sufficient cell numbers for progressing towards cell therapy approaches. In Table 1, we summarise the relevant modification of the MHH and UCL protocols from the original protocol by Kim et al., 2017 [25].

However, the long-term viability of hiPSC-SCs in vitro is still a tricky problem, which would greatly limit the prospect of their clinical use. Therefore, we still need to explore how to improve the survival and proliferation of hiPSC-SCs in vitro, before the application of hiPSC-derived hiPSC-SCs in cell therapy approaches could make a difference in the treatment of peripheral neuropathies.

## Figures and Tables

**Table 1:** Comparison of three differentiation protocols.

	D0-D6	D6-D24	D24-D26	D26-D28	>D30
Kim et al. 2017 [25]	D0 = hiPSC	D24 + = hiPSC-SCP			D30 + = hiPSC-SC
	Matrigel coating				
	Neurobasal/Advanced DMEM/F12 N2, B27, SB431542, CHIR99021, BSA, β-ME		DMEM (1 g/L glucose) 1% FBS, NRG1		
		NRG1	RA, FSK, PDGF-BB	PDGF-BB	
MHH-Protocol	D0 = hiPSC	D24 + = hiPSC-immatureSC			D30 + = hiPSC-SC
	Matrigel coating			P-ORN-laminin coating	
	Neurobasal/Advanced DMEM/F12 N2, B27, SB431542, CHIR99021, BSA, β-ME, Y27632, Pen/Strep, Amphotericin B		Advanced DMEM/F12 (4.5 g/L glucose) 10% FBS, Pen/Strep, Amphotericin B, NGR1		ahSC medium
		NRG1	RA, FSK, PDGF-BB	PDGF-BB	
		Cells dissociated when reaching 60–70% confluence	Seeding density 21,052 cells/cm^2^	Seeding density 42,104 cells/cm^2^	
UCL-Protocol	D0 = hiPSC	D24 + = hiPSC-SCP			D30 + = hiPSC-SC
	Geltrex^TM^ matrix				
	Neurobasal/Advanced DMEM/F12 N2, B27, SB431542, CHIR99021, BSA, β-ME		DMEM(1 g/L glucose) 1% FBS, NRG1		
		NRG1	RA, FSK, PDGF-BB	PDGF-BB	
		Cells dissociated when reaching 90% confluence	Seeding density 14,104 cells/cm^2^		

D: day of differentiation; hiPSC: human induced pluripotent stem cell; hiPSC-SCP: human iPSC-derived Schwann cell precursor; hiPSC-immatureSC: human iPSC-derived immature Schwann cell; hiPSC-SC: hiPSC-derived Schwann cell; blue and yellow background colour indicate type of coating; magenta background and white font colour indicate relevant modifications from the original protocol [25]; β-ME: β-mercaptoethanol; FBS: foetal bovine serum; FSK: forskolin; NRG1: neuregulin 1; BSA: bovine serum albumin; RA: retinoic acid; PDGF-BB: platelet-derived growth factor-bb; ahSC medium: melanocyte growth medium, fibroblast growth factor, bovine pituitary extract, FSK, insulin, NRG1, Pen/Strep, amphotericin B [61].

## Data Availability

The datasets analysed during this study are available from the corresponding authors on request. Raw data are stored in the authors’ institutional repositories and will be provided upon request accordingly.

## Supplementary Materials

The following supporting information can be downloaded at: https://www.mdpi.com/article/10.3390/ijms24065366/s1.
