# Supplementary material for: Culture Conditions for Human Induced Pluripotent Stem Cell-Derived Schwann Cells: A Two-Centre Study
Source: Int J Mol Sci. 2023 Mar 10;24(6):5366. doi: 10.3390/ijms24065366 (PMC10049204; doi:10.3390/ijms24065366)
Supplement: Supplementary file 1 [file ijms-24-05366-s001.zip › ijms-2194610-supplementary.pdf]

International Journal of *Molecular Sciences*

Article

Culture Conditions for Human Induced Pluripotent Stem Cell-Derived Schwann Cells: A Two-Centre Study

Zhong Huang [1,2,†], Rebecca Powell [3,†], Svenja Kankowski [1], James B. Phillips [3,4,*,‡] and Kirsten Haastert-Talini [1,2,*,‡]

1 Institute of Neuroanatomy and Cell Biology, Hannover Medical School (MHH), 30623 Hannover, Germany
2 Center for Systems Neuroscience (ZSN) Hannover, 30559 Hannover, Germany
3 Department of Pharmacology, University College London (UCL) School of Pharmacy, 29-39 Brunswick Square, London WC1N 1AX, UK
4 UCL Centre for Nerve Engineering, UCL, London WC1H 0AL, UK
* Correspondence: jb.phillips@ucl.ac.uk (J.B.P.); haastert-talini.kirsten@mh-hannover.de (K.H.-T.)
† These authors contributed equally to this work.
‡ These authors contributed equally to this work.

Supplementary Material

Table S1. Primary and secondary antibodies for immunocytochemistry at MHH; Table S2. Primary and secondary antibodies for immunocytochemistry at UCL; Table S3. MHH primer sequences for RT-Qpcr; Table S4. UCL primer sequences for RT-qPCR.

Figure S1. Characterization of hiPSC-SCPs morphology, mRNA and protein level when cultured in different cell densities and in different passages; Figure S2. Characterization of hiPSC-SCPs morphology, mRNA and protein level when cultured in different cell densities and in different passages; Figure S3. Microscopic comparison of hiPSC-SCs derived from the original and the MHH modified protocol; Figure S4. Phase contrast micrographs showing morphological change between high-density hiPSC-SCs, low density hiPSC-SCs and ahSCs; Figure S5. Phase contrast micrographs showing hiPSC-SCPs plating density prior to hiPSC-SC differentiation and hiPSC-SC survival after 7 days; Figure S6. Morphology of hiPSC-SCs at DIV 15 in SCDM.

International Journal of
Molecular Sciences

MDPI

Table S1. Primary and secondary antibodies for immunocytochemistry <u>*at MHH*</u>.

**Antibodies for immunocytochemistry**					
Antibodies	Dilution	Species Raised	Product code	Source	Epitope
GAP43	1:500	Rabbit polyclonal	AB5220	Abcam	Cytoplasm
CDH 19	1:200	Rabbit polyclonal	PA5-101291	Invitrogen	Cytoplasm
SOX10	1:100	Mouse monoclonal	MAB2864	R&D	Nucleus/ Cytoplasm
$p75^{NTR}$	1:200	Mouse monoclonal	AB3125	Abcam	Extracellular
S100B	1:200	Rabbit polyclonal	Z0311	Dako	Cytoplasm
GFAP	1:400	Rabbit polyclonal	G-9269	Sigma	Cytoplasm
GALC	1:500 or 1:200	Rabbit polyclonal	G9152	Sigma	Extracellular

Table S2. Primary and secondary antibodies for immunocytochemistry <u>*at UCL*</u>.

**Antibodies for immunocytochemistry – second modified protocol**					
Antibodies	Dilutio n	Species Raised	Product code	Source	Epitope
OCT4	1:200	Goat polyclonal	sc-8629	Santa Cruz Biotechnology	Nucleus
SOX10	1:40	Mouse monoclonal	sc-365692	Santa Cruz Biotechnology	Nucleus/ Cytoplasm
$p75^{NTR}$	1:200	Rabbit monoclonal	8238	Cell Signalling Technology	Extracellular
S100B	1:1	Rabbit polyclonal	GA50461-2	Dako	Cytoplasm
DAPI	1:1000	Fluorescent dye	D9542	Sigma	Nucleus
DyLight 488 Anti-Mouse IgG	1:200	Horse polyclonal	DI-2488	VectorLabs	Secondary
DyLight 549 Anti-Rabbit IgG	1:200	Goat polyclonal	DI-1549	VectorLabs	Secondary
DyLight 594 Anti-Goat IgG	1:200	Horse polyclonal	DI-3094	VectorLabs	Secondary

Table S3. MHH primer sequences for RT-qPCR.

Gene	Forward primer (5′ to 3′)	Reverse primer (5′ to 3′)	Expected amplicon size (bp)
RPLP0	TGGTCTCTTTGACTAATCACC	AGAAGTAAGCCTTTATTTCCTT	80
SNAI2	CTGGACACACATACAGTGATTA	CGGTAGTCCACACAGTGAT	121
TFAP2A	GCAGTAGCTGAATTTCTCAAC	CTCTTTGCATATCTGTTTTGTA	96
CDH19	GTAATAGACATCGCTACTGGAA	GATAACTAATGTTCCTTCTGGA	15-
NGFR	ACAACCTCATCCCTGTCTAT	AGCTGTTCCACCTCTTGA	87
S100B	GAGACAAGGAAGAGGATGTC	TAAGAAATGGGAAAGCTCAT	150
PMP22	AAATTCTTGCTGGTCTGTG	GTAGGAGTAATCCGAGTTGAG	89
SOX10	GAGAGGGCTCCCCCATGTCAGA	GCCCGACTGCAGCTCTGTCTTC	104
c-Jun	ACAATAGGTGCTTATTCTCAAA	CTAGGAATTGTCAAAGAGAAGA	94
PLP1	GAGAAAAAGTAAAAGACCGAAG	ACTCTAACAAGCCCATGTC	135
GAP43	CTGAAGAGAACATAGAAGCTGT	AAAGCCATTTCTTAGAGTTCA	122

Table S4. UCL primer sequences for RT-qPCR.

Gene	Forward primer (5′ to 3′)	Reverse primer (5′ to 3′)	Expected amplicon size (bp)
RPS18	CAAGAGGGCGGGAGAAC	CGTGGATTCTGCATAATGGTG	66
TBP	ACTTCGTGCCCGAAACG	GTGGTTCGTGGCTCTCTTATC	78
SNAI2	CCAAACTACAGCGAAC	TGAGGATCTCTGGTTG	97
TFAP2A	GAAGCTGTCCACCTAGC	CTTTGGCAGGAAATTCGG	61
CDH19	CTTGTCTTGGAGCAACAG	ATCTTAGCTGGCCGATG	150
NGFR	GTGAGTGCTGCAAAG	AACGTCACGCTGTC	97
S100B	CTCATCAACAATGAGCTTTC	TCACATTCGCCGTCTC	104
PMP22	GACACGCAACTGATCTC	TGCAGCCATTCGTTTG	91
SOX10	TACACCGACCAGCCATC	GGTCAGAGTAGTCAAACTGG	109
c-Jun	CCTGATAATCCAGTCC	ATCTGTCACGTTCTTG	82
PLP1	ACCTGCCAGTCTATTG	TGGGAGAACACCATAC	87
GAP43	AGCTCATAAGGCCGCAAC	TCAGCAGCTTGGACATCATC	99
SOX2	GCTCGCAGACCTACATGAAC	ACTTGACCACCGAACCC	102

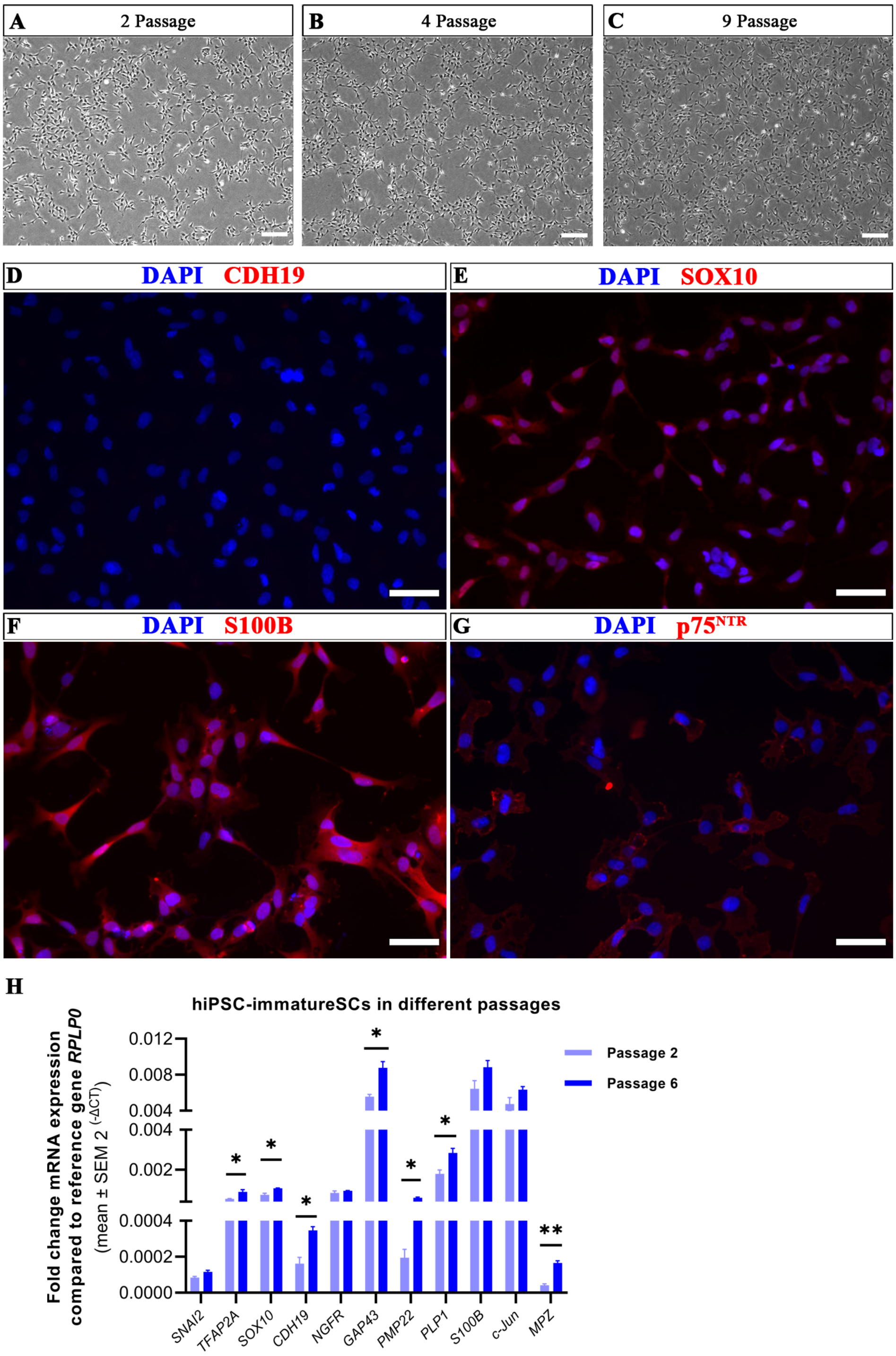

Figure S1. Characterization of MHH hiPSC-immatureSCs morphology, mRNA and protein level when cultured in different passages. (A-C) Representative phase-contrast photomicrographs of cells cultured at passage 2, 4, and 9 with an initial cell density of 2×10⁵ . (D-G) Immunocytochemical characterization of hiPSC-immatureSCs from passages 2-9, cells are negative for CDH19 (D) and positive for Schwann cell markers SOX10 (E), S100B (F), and $p75^{NTR}$ (G); cell nuclei were stained with DAPI (blue). (H) Graphical presentation of gene expression analysis of hiPSC-immatureSCs at passage 2 and passage 6 with gene *SNAI2, TFAP2A, SOX10, CDH19, NGFR, GAP43 PMP22, PLP1, S100B, c-Jun*, and *MPZ*. Values were normalized to the reference gene *RPLP0*; Statistical analysis with unpaired t-test, * $P < 0.05$, ** $P < 0.01$, $n = 3$ cell cultures. (A-C) scale bar: 200 µm. (D-G) Scale bar: 50 µm.; hiPSC-immatureSCs = human induced Pluripotent Stem Cell derived immature Schwann Cells.

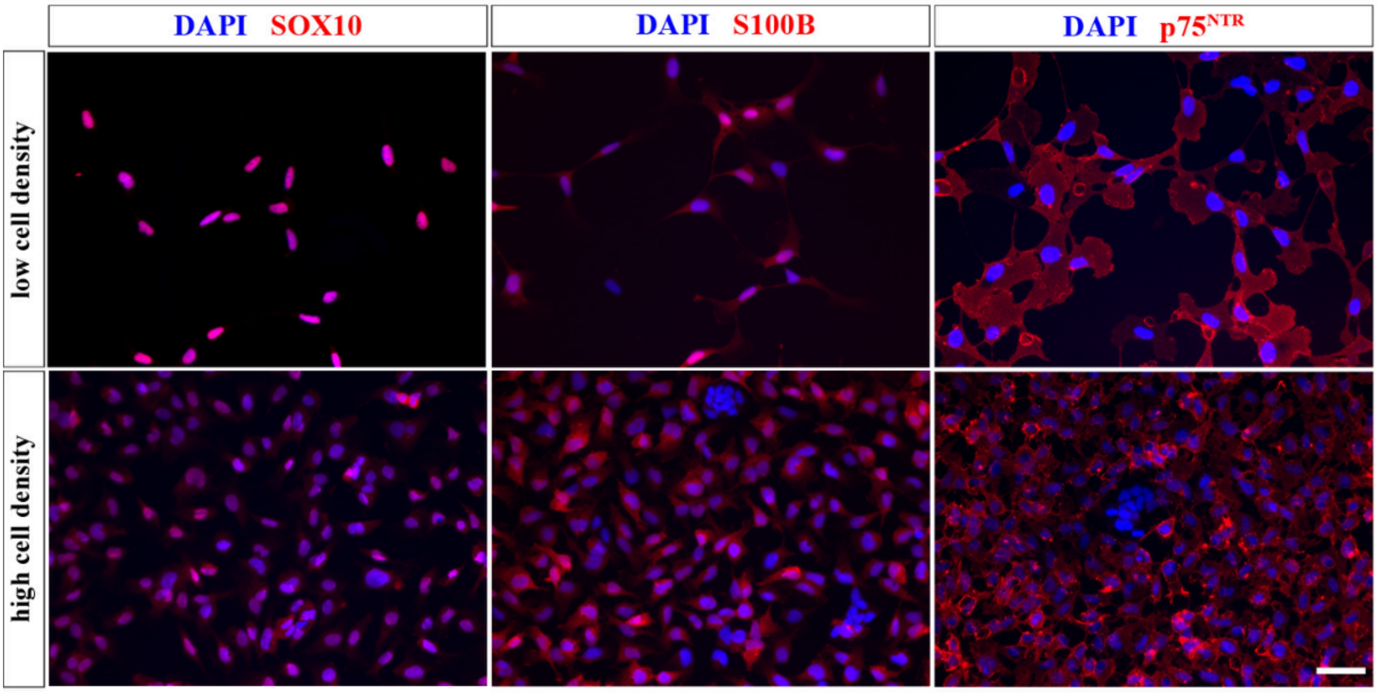

Figure S2. Morphology changes of hiPSC-immatureSCs cultured with different cell densities. Low cell density stands for a seeding density of 2.5 x 10^4 cells / 24-well, and high cell density stands for a seeding density of 5 x 10^4 cells / 24-well. The cells in both cultures were immunopositive for SOX10, S100B, and $p75^{NTR}$. Cell nuclei were stained with DAPI (Blue). Scale bar: 50 µm. hiPSC-immatureSCs = human induced Pluripotent Stem Cell derived immature Schwann Cells.

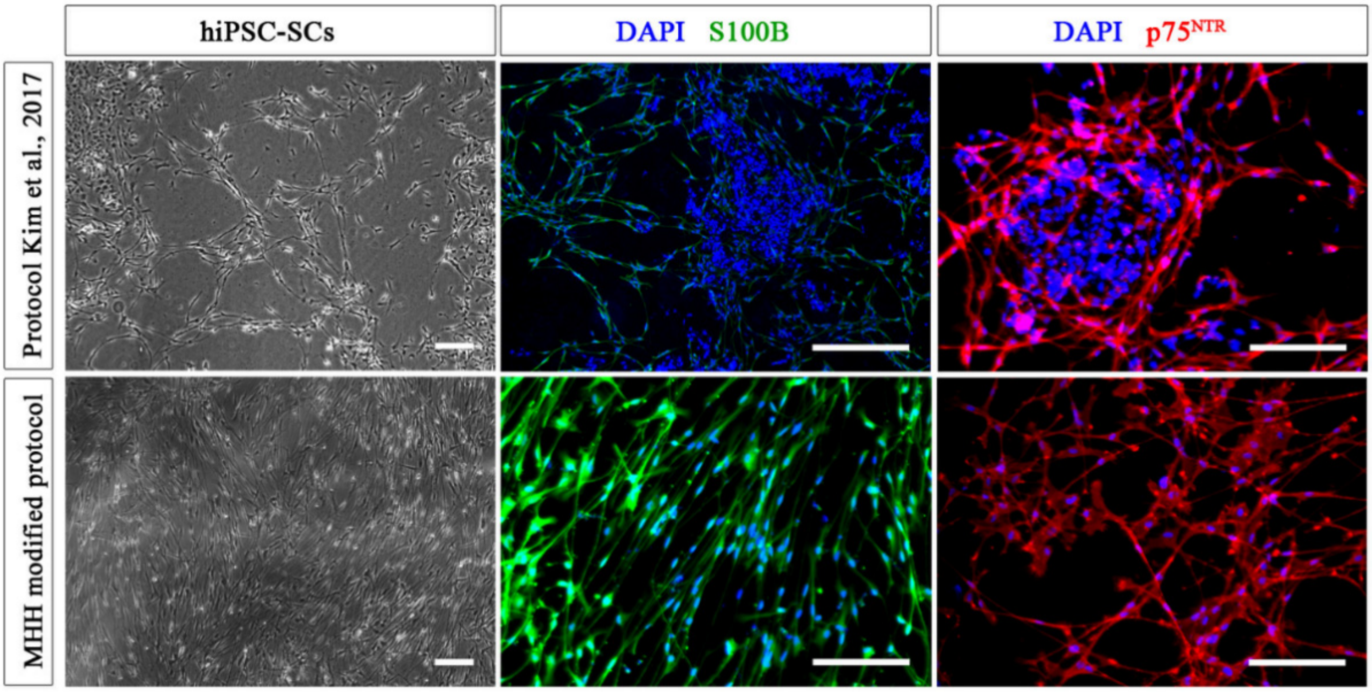

Figure S3. Microscopic comparison of hiPSC-SCs derived from the original and the MHH modified protocol. Phase-contrast photomicrographs represent hiPSC-SCs differentiated by using the unmodified protocol according to [25] and the MHH modified

protocol. Immunocytochemical staining was performed to detect Schwann cell markers S100B and p75^{NTR}. Cell nuclei were stained with DAPI (Blue). Scale bar: 200 µm. hiPSC-SCs = human induced Pluripotent Stem Cell derived Schwann Cells.

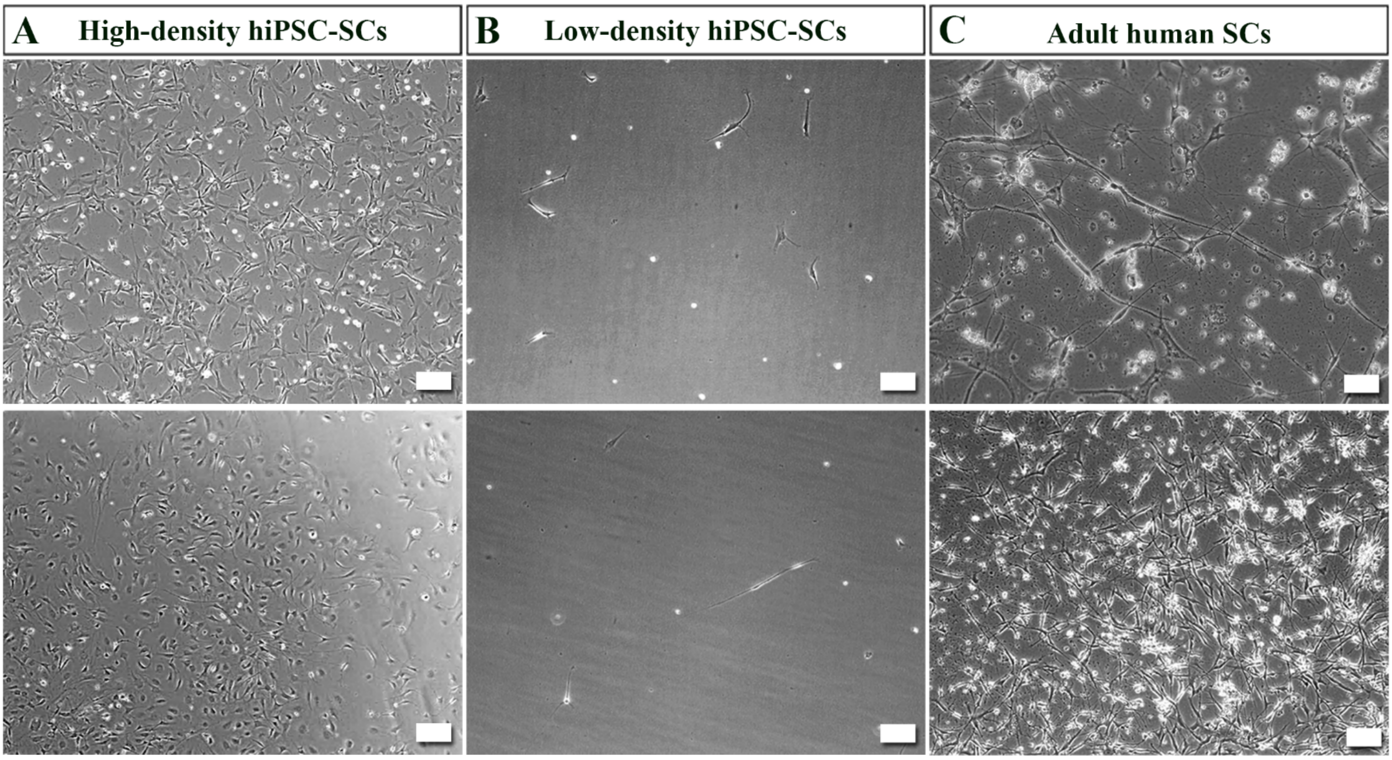

Figure S4. Phase contrast micrographs showing morphological change between high-density hiPSC-SCs (A), low-density hiPSC-SCs (B) and adult human Schwann cells cultured at UCL (C). Low cell density stands for a seeding density of 1763 cells / cm^2, and high cell density stands for a seeding density of 14101 cells / cm^2. Adult human Schwann cells in (C) were seeded in a density of 3.7 x 10^4 cells / cm^2. Upper and lower panels show examples from two differentiation procedures for the differentiated Schwann cells while the adult human Schwann cells images are from two separate flasks from one cell line. Low density plating produces cells more closely resembling the primary Schwann cell culture, being longer and more bipolar in shape. Scale bar = 100 µm. hiPSC-SCs were differentiated from hiPSC-SCPs for 7 days in SCDM. hiPSC-SCPs = human induced Pluripotent Stem Cell derived Schwann Cell Precursor cells; hiPSC-SCs = human induced Pluripotent Stem Cell derived Schwann Cells.

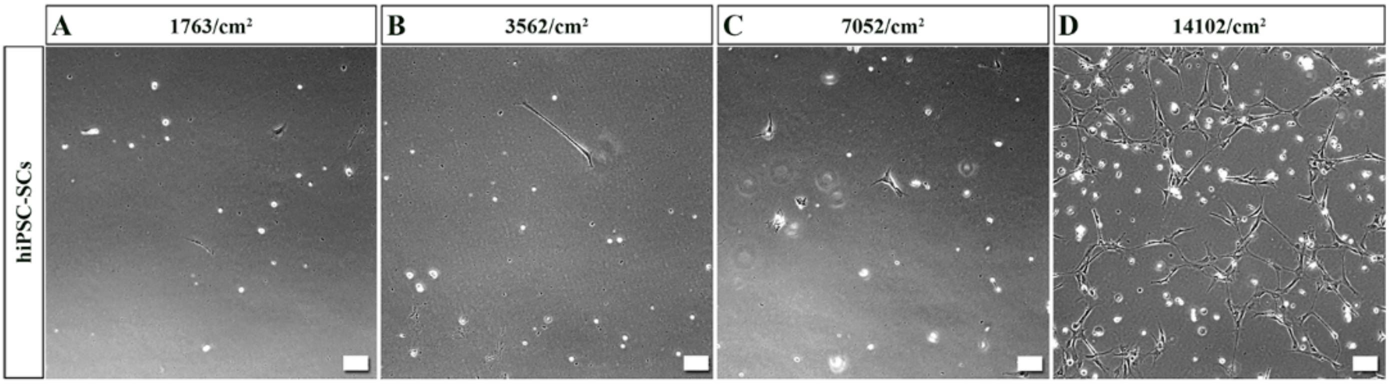

Figure S5. Phase contrast micrographs showing UCL hiPSC-SCPs plating density prior to hiPSC-SC differentiation and hiPSC-SC survival after 7 days. (A) 1763/cm²; (B) 3526/cm²; (C) 7052/cm²; (D): 14102/cm²; Scale bar = 100µm. Survival of hiPSC-SCs may be increased by increasing plating density of hiPSC-SCPs 8-fold. hiPSC-SCPs = human induced Pluripotent Stem Cell derived Schwann Cell Precursor cells.

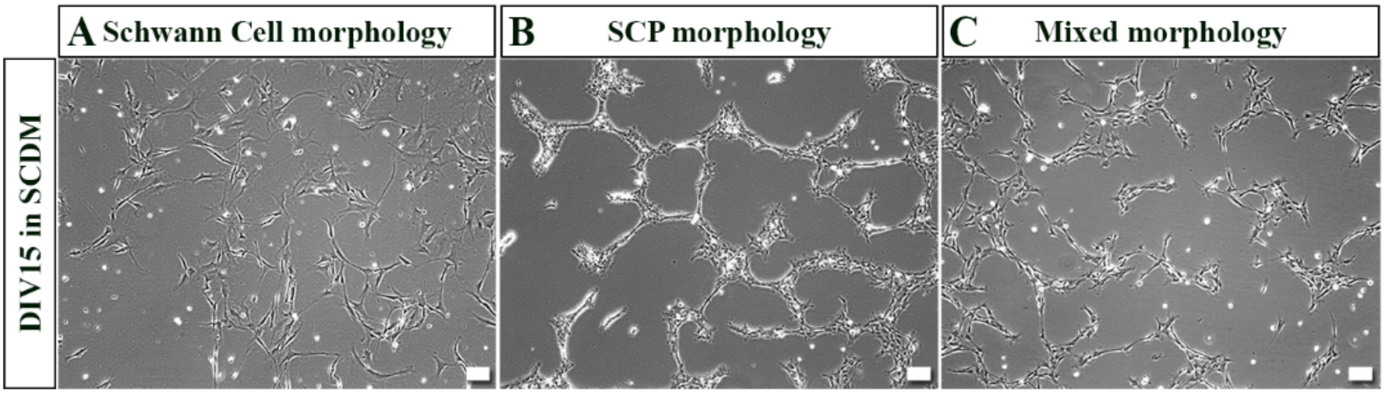

Figure S6. Morphology of UCL hiPSC-SCs at DIV 15 in UCL-SCDM. Differing morphologies can be seen with the long, bipolar morphology seen in Schwann cells evident in (A). (B) shows cells closer in morphology to the hiPSC-SCPs (dense, small and closely associated with one another) while (C) shows a mixture of these cell types. The changes in gene expression seen between DIV 7 and DIV 15 in figure 9 (RT-qPCR results) could be due to mixed populations of cell types appearing by DIV 15. UCL-SCDM = Schwann cell differentiation medium as used by the UCL team. Scale bar = 100µm.
